# Linking Pedigree Information to the Gene Expression Phenotype to Understand Differential Family Survival Mechanisms in Highly Fecund Fish: A Case Study in the Larviculture of Pacific Bluefin Tuna

**DOI:** 10.3390/cimb43030145

**Published:** 2021-11-26

**Authors:** Motoshige Yasuike, Kazunori Kumon, Yosuke Tanaka, Kenji Saitoh, Takuma Sugaya

**Affiliations:** 1 Bioinformatics and Biosciences Division, Fisheries Stock Assessment Center, Fisheries Resources Institute, Japan Fisheries Research and Education Agency, 2-12-4 Fuku-ura, Kanazawa, Yokohama 236-8648, Kanagawa, Japan; 2 Amami Field Station, Tuna Aquaculture Division, Aquaculture Research Department, Fisheries Technology Institute, Japan Fisheries Research and Education Agency, 955-5 Hyousakiyamahara, Setouchi 894-2414, Kagoshima, Japan; kumon@affrc.go.jp; 3 Highly Migratory Resources Division, Fisheries Stock Assessment Center, Fisheries Resources Institute, 5-7-1 Orido, Shimizu-Ku, Shizuoka-Shi 424-8633, Shizuoka, Japan; yosuket@affrc.go.jp; 4 Shiogama Branch, Fisheries Resources Institute, Japan Fisheries Research and Education Agency, Shinhama 3-27-5, Shiogama 985-0001, Miyagi, Japan; ksaitoh@affrc.go.jp; 5 Momoshima Field Station, Production Engineering Division, Fisheries Technology Institute, Japan Fisheries Research and Education Agency, 1760 Momoshima, Onomichi 722-0061, Hiroshima, Japan; tsugaya@affrc.go.jp

**Keywords:** gastric dysfunction, carnivorous fish, piscivory, larval mortality, energy budget, diet shift, fish farming, oligonucleotide microarray

## Abstract

Mass spawning in fish culture often brings about a marked variance in family size, which can cause a reduction in effective population sizes in seed production for stock enhancement. This study reports an example of combined pedigree information and gene expression phenotypes to understand differential family survival mechanisms in early stages of Pacific bluefin tuna, *Thunnus orientalis*, in a mass culture tank. Initially, parentage was determined using the partial mitochondrial DNA control region sequence and 11 microsatellite loci at 1, 10, 15, and 40 days post-hatch (DPH). A dramatic proportional change in the families was observed at around 15 DPH; therefore, transcriptome analysis was conducted for the 15 DPH larvae using a previously developed oligonucleotide microarray. This analysis successfully addressed the family-specific gene expression phenotypes with 5739 differentially expressed genes and highlighted the importance of expression levels of gastric-function-related genes at the developmental stage for subsequent survival. This strategy demonstrated herein can be broadly applicable to species of interest in aquaculture to comprehend the molecular mechanism of parental effects on offspring survival, which will contribute to the optimization of breeding technologies.

## 1. Introduction

Early life stages in many fish species are characterized by high mortality. Fecundity of these fishes is accordingly big, and they typically broadcast their eggs through mass spawning [1]. When culturing these highly fecund fishes, mass spawning arbitrarily in a single tank or cage is a common practice [2]. This reproductive strategy often exhibits a highly imbalanced family structure in offspring, as revealed by genetic-marker-based parentage analyses in a number of aquaculture species, and thereby can lead to a high risk of rapid gene loss [3]. In an extreme case involving the Japanese flounder, *Paralichthys olivaceus*, more than 99% of larvae from a mass spawning event were sired by a single male in a cohort of six males [4]. Causes of highly variable family survival seem to vary across species, which are influenced by developmental stages, feed formulation, aquaculture facilities, and environmental factors. A better understanding of the mechanisms behind differential family survival will provide valuable information for improving breeding technology in aquaculture.

The genetic basis of natural variation in gene expression exists in organisms from yeast to humans [5], including fish [6,7]. This phenotypic trait, i.e., the gene expression phenotype, also exhibits familial aggregation and a heritable component [8,9,10,11,12]. Thus, linking pedigree information to the gene expression phenotype may provide clues for understanding the molecular mechanism underlying differential family survival in larviculture of aquaculture species.

To test this hypothesis, this study investigated the parental effects on survival and the gene expression phenotype in early stages of Pacific bluefin tuna (PBT), *Thunnus orientalis*, which were developed in a mass culture tank. Although in 2002, full-life-cycle aquaculture of PBT was developed at Kindai University in Japan, heavy mortality in the larviculture of PBT has been observed [13,14]. We initially examined parentage and family representation for progenies of PBT derived from the mass spawning of a broodstock group at 1, 10, 15, and 40 days post-hatch (DPH) in a mass culture tank. A remarkable degree of proportional change in the family representations occurred after 15 DPH; therefore, we performed transcriptome analysis of the 15 DPH larvae using a previously developed 44 K PBT oligonucleotide microarray (oligo-array) [15]. The analysis successfully addressed the causes of differential family survival by gene expression phenotypes.

## 2. Materials and Methods

### 2.1. Parentage Assessment

**Figure 1:**
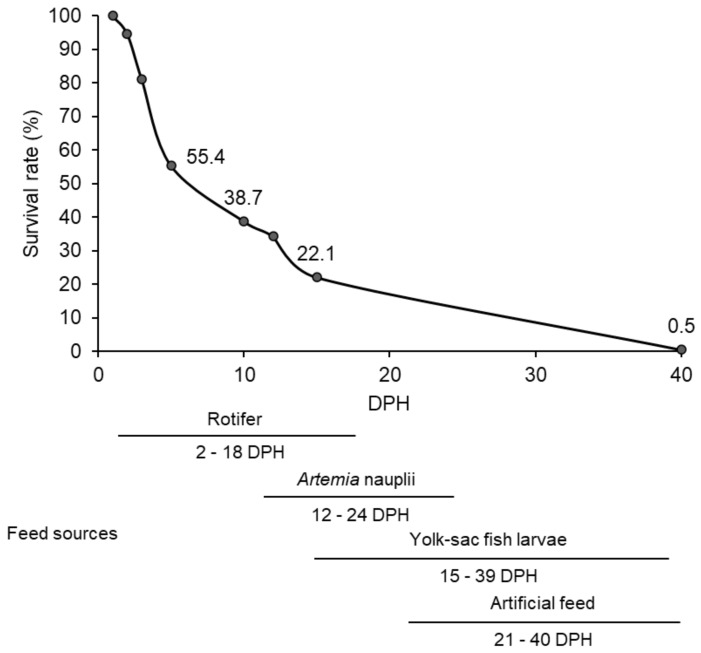
Survival rate of hatchery-reared Pacific bluefin tuna larvae in a hatchery tank. DPH indicates days post-hatch. Food sources are also shown below the graph.

Fertilized eggs of PBT were obtained on August 31, 2013, from spontaneous spawns of broodstock (7 years old, *n* = 71) derived from wild-caught yearling tuna in a 40-m-diameter net pen at the Amami Field Station, Fisheries Research and Education Agency. Fertilized eggs were kept in a 500 L tank until 1 DPH, and the larvae at 1 DPH were transferred into a 50 kL mass culture tank (11,100 individuals per m^3^, water temperature 27–28 °C). Larvae and juveniles were fed the following: rotifer (*Brachionus plicatilis* sp. complex, 10 individuals per cc) from 2 to 18 DPH, *Artemia* nauplii from 12 to 24 DPH, yolk-sac larvae of the spangled emperor (*Lethrinus nebulosus*) from 15 to 39 DPH, and an artificial feed from 21 to 40 DPH (Figure 1). For parentage analysis, a random sample of whole larvae at 1, 10, 15, and 40 DPH (100–500 individuals each) was collected in 100% ethanol and stored at −20 °C until genomic DNA (gDNA) extraction. In addition, larvae of 15 DPH (200 individuals) were also preserved in RNAlater stabilized solution (Thermo Fisher Scientific, Waltham, MA, USA) for both parentage assessment (gDNA) and transcriptome analysis (RNA). The samples in RNAlater were stored at −20 °C until use. Next, 15 DPH larvae (96 individuals) were divided with a scalpel into the caudal portion for gDNA samples and the rest of the body, including the head and internal organs, for total RNA extraction. Genomic DNA samples were extracted from a random sample of 96 progenies at each sampling point using the QuickGene system (Kurabo, Neyagawa, Japan). In contrast, extraction of gDNA from broodstock was performed using a fin clip. Double-stranded sequencing of a region of mitochondrial DNA (mtDNA) encompassing the 3′ portion of cytochrome b to the left domain of the control region determined maternal lines (position 15,246 to 16,156 of a complete mtDNA sequence of *Thunnus orientalis* (AB185022)). The primers used for PCR and sequencing were CBCR-LT (CAC ATT AAA CCT GAA TGA TA) and MTCR-R1 (CAT TAT TGT ATT TGC ACT GTG A). The PCR profile has been described in another study [16]. PCR products were sequenced from both ends using the BigDye Terminator v.3.1 kit run on an ABI3730 sequencer (ABI, Foster City, CA, USA). Parentage analysis was performed based on the mtDNA control region and 11 microsatellite (MS) loci using the procedure previously described [17].

### 2.2. Transcriptome Analysis

Here, 4 individuals from each of the 15 DPH larvae from four full-sib families (16 samples) were used for transcriptome analysis. Total RNA was isolated using the RNeasy Plus Universal Mini Kit (Qiagen, Inc., Valencia, CA, USA), and samples were treated with 2 units of TURBO DNase from the TURBO DNase-free Kit (Life Technologies, Carlsbad, CA, USA). Transcriptome analysis was performed using a 44 K PBT oligo-array. Details of the oligo-array and experimental procedures have been described in another study [15]. Oligo-array experiments were performed using an Agilent One-Color platform following the One-Color Microarray-Based Gene Expression Analysis protocol version 6.5 (Agilent Technologies, Santa Clara, CA, USA). Oligo-array slides were scanned on an Agilent DNA Microarray Scanner with Surescan High-Resolution Technology (Agilent Technologies, Santa Clara, CA, USA), and features were extracted and quantified from scanned images using Feature Extraction software version 10.7.3.1 (Agilent Technologies, Santa Clara, CA, USA). Normalization and differential expression analyses were performed using GeneSpring version 12.6 (Agilent Technologies, Santa Clara, CA, USA). After 75th percentile shift normalization, the differentially expressed genes (DEGs) were considered significant according to one-way analysis of variance (ANOVA) followed by a Benjamini–Hochberg multiple testing correction and Tukey HSD post hoc test with a corrected *p*-value cut-off of 0.05 and a change of two-fold or greater in at least one full-sib family. Visualization of the DEGs was performed by constructing a hierarchical heatmap with Euclidean distance and complete linkage using GeneSpring version 12.6 (Agilent Technologies, Santa Clara, CA, USA).

### 2.3. RT-qPCR Assay

To validate oligo-array results, we performed an RT-qPCR assay for three pepsinogen genes (*PG1*, *PG2*, and *PG3*), two potassium-transporting ATPase subunit genes (*ATP4A* and *ATP4B*), and the β-actin gene (internal control) using the same RNA samples from the oligo-array analysis. PCR primers and FAM™ dye-labeled TaqMan^®^ minor groove binder probes (Applied Biosystems, Foster City, CA, USA) are shown in Table 1.

cDNA was synthesized from 1 μg of total RNA using the PrimeScript™ RT reagent kit with gDNA Eraser (Takara, Otsu, Japan). RT-qPCR was performed using Premix Ex Taq™ (Probe qPCR; Takara, Otsu, Japan) in a Thermal Cycler Dice^®^ Real-time System TP800 (Takara, Otsu, Japan) using the following program: 95 °C for 30 s, 40 cycles of 95 °C for 5 s, and 60 °C for 30 s. The relative expression level of each target gene was calculated using the 2^−ΔΔC^_T_ method [18] and normalized to the expression of the β-actin gene. The qPCR data were analyzed using Welch’s *t*-test, and differences were considered statistically significant at *p* < 0.05.

An RT-qPCR assay for the PG2 gene was conducted for all genotyped 15 DPH progenies (96 individuals) by an absolute quantification method using standard curves derived from serial dilutions of the plasmid standard encoding the target sequence. cDNA synthesis and qPCR procedures were the same as above. Normalization of values was performed by dividing the number of copies of the PG2 gene by that of the β-actin gene. Statistical significance (*p* < 0.05) was determined by one-way ANOVA with Tukey post hoc analysis (among full-sib families) or by the Wilcoxon rank-sum test (between progenies of two females).

## 3. Results

### 3.1. The Influence of Parental Genetic Effects on Offspring Survival

**Figure 2:**
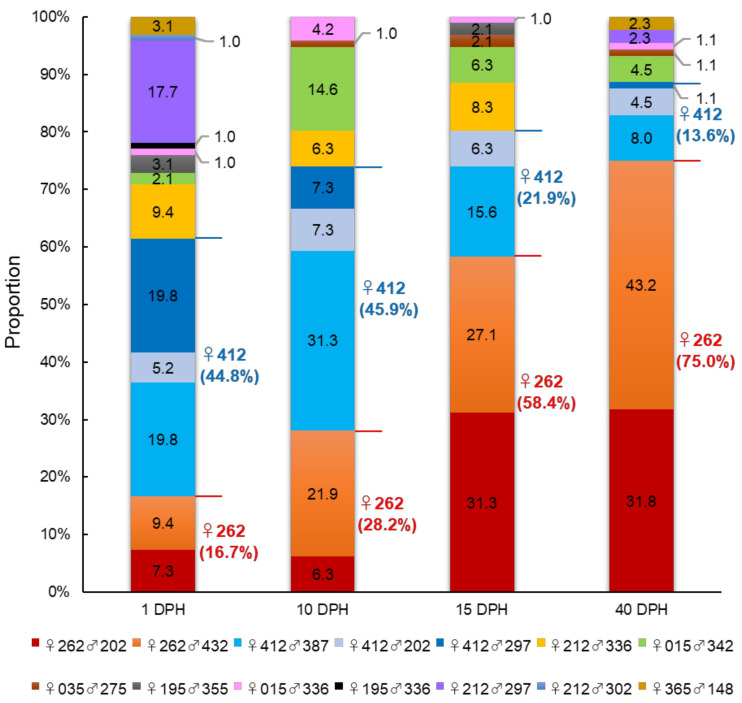
Parentage assignment for Pacific bluefin tuna larvae at 1, 10, 15, and 40 days post-hatch (DPH) using 11 microsatellite (MS) markers.

A total of 44.4 × 10^4^ larvae at 1 DPH were transferred into a 50 kL mass culture tank. Survival rates in the hatchery tank decreased sharply to 22.1% by 15 DPH (Figure 1). Finally, the survival rate was 0.5% at 40 DPH (Figure 1). A total of 376 progenies (96 each from 1, 10, and 15 DPH and 88 from 40 DPH) were successfully assigned to their parents. Among them, 14 full-sib families were generated by 7 females and 10 males. Although the family size was unequal at 1 DPH, a large variance in family size was observed dramatically after 15 DPH (Figure 2). In particular, ♀412 was the highest contributing dam at 1 DPH (44.8%) and 10 DPH (45.9%), but the contribution considerably decreased at 15 DPH (21.9%) and 40 DPH (13.6%) (Figure 2). In contrast, the contribution of ♀262 was much lower than that of ♀412 at 1 DPH (16.7%) and 10 DPH (28.2%), but the contribution dramatically increased at 15 DPH (58.4%) and 40 DPH (75.0%) (Figure 2). Families ♀262♂202 and ♀412♂202 shared the same sire (♂202), and the proportion of ♀262♂202 (31.8%) was 7-fold higher than that of ♀412♂202 (4.5%) at 40 DPH (Figure 2). Therefore, these results showed that the effect of dams on post-hatching survival is larger than that of sires, and dam ♀262 had a strong positive effect on offspring survivability during development. In addition, in ♀412-dammed families, a sire effect was also observed for ♂202, as the contribution was nearly unchanged from 5.2% (1 DPH) to 4.5% (40 DPH), while the other sibs in this maternal line markedly decreased from 19.8% (1 DPH) to 1.1% (40 DPH) for ♂297 and 19.8% (1 DPH) to 8.0% (40 DPH) for ♂387 (Figure 2).

### 3.2. Parental Effect on Gene Expression Phenotype

**Figure 3:**
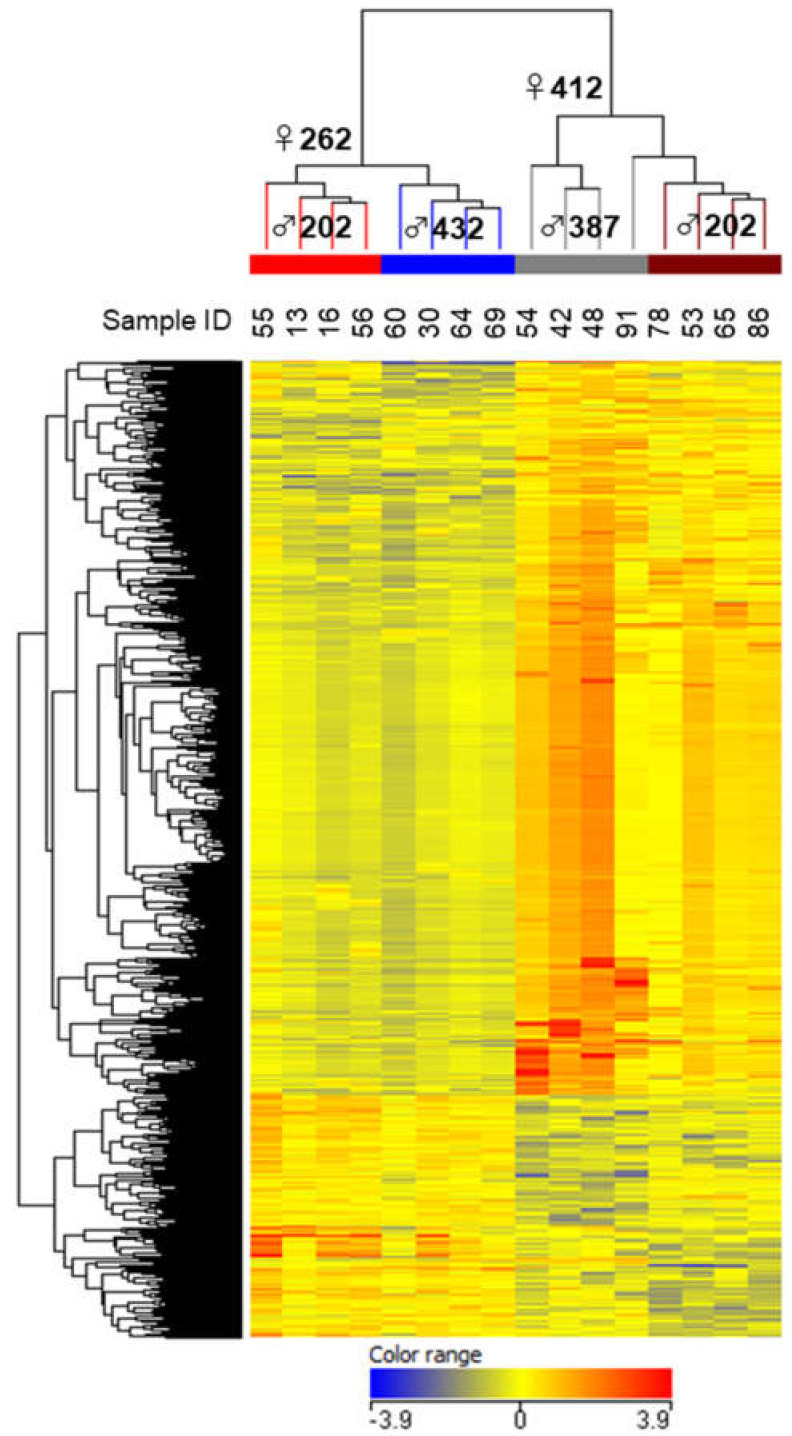
Hierarchically clustered heat map of 5739 differentially expressed genes (DEGs) among four full-sib families, ♀262♂202, ♀262♂432, ♀412♂387, and ♀412♂202 (four individuals each), of the 15 DPH Pacific bluefin tuna larvae.

Because a considerable change in family representation was observed after 15 DPH, transcriptome analysis was conducted for the PBT larvae at 15 DPH. Based on the parentage assessment (Figure 2), we chose four full-sib families (four individuals each), ♀262♂202, ♀262♂432 (increased family size), ♀412♂387 (decreased family size), and ♀412♂202 (nearly unchanged and small family size), for transcriptome analysis. The hierarchically clustered heat map clearly exhibited family-specific gene expression patterns with 5739 DEGs (Figure 3). In particular, the gene expression patterns were divided into two large clusters by dams: ♀262 and ♀412 (Figure 3). Thus, these results indicate the parental effect on the gene expression phenotype of offspring and a stronger maternal effect compared with the paternal effect at 15 DPH.

**Figure 4:**
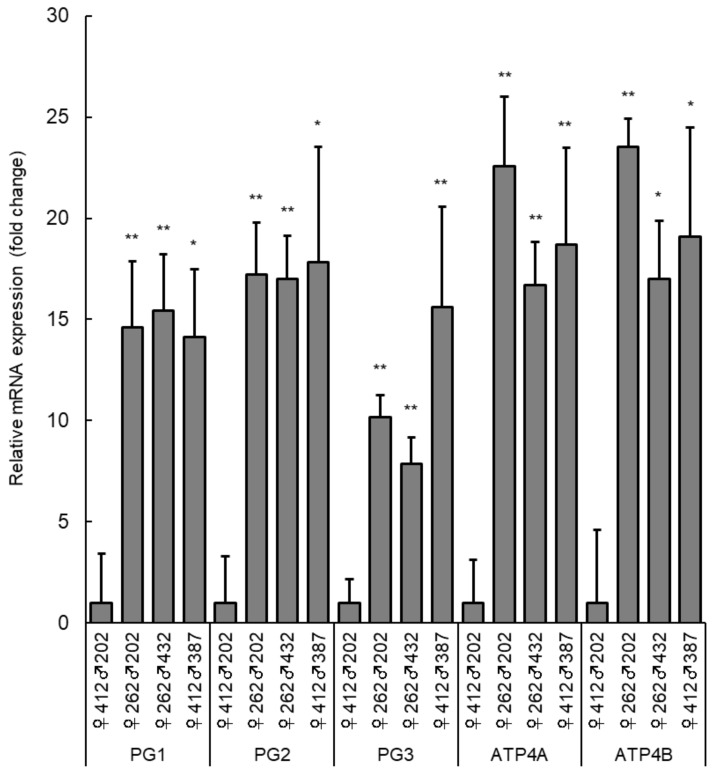
RT-qPCR analysis of the highly expressed genes, three pepsinogen genes (*PG1*, *PG2*, and *PG3*) and two potassium-transporting ATPase subunit genes (*ATP4A* and *ATP4B*), in three families (♀262♂202, ♀262♂432, and♀412♂387) of the 15 DPH Pacific bluefin tuna larvae. The graph represents the relative fold-change values when compared to the ♀412♂202 family, and error bars show the standard error of the mean (SEM). Asterisks indicate a statistically significant difference (* *p* < 0.05; ** *p* < 0.01) when compared with the high-mortality family (♀412♂202).

The gene expression profiles of three families (♀262♂202, ♀262♂432, and ♀412♂387) were compared with those of the nearly unchanged family, ♀412♂202. The top 20 most highly expressed genes in the three families are listed in Table 2. Notably, the acid-secretion-related genes, *ATP4A* and *ATP4B*, and pepsinogen genes, *PG1*, *PG2*, and *PG3*, were highly expressed in ♀262-dammed families and ♀412♂387, with more than a 10-fold difference (Table 2). These results were confirmed by reverse transcription–quantitative polymerase chain reaction (RT-qPCR; *n* = 4 for each family; Figure 4). ATP4A and ATP4B are subunits of H^+^/K^+^-ATPase, which is a gastric proton pump that facilitates the acidic environment necessary for acid hydrolysis of food in the stomach [19,20]. Pepsinogen is the major acidic protease in the stomach, which activates the active form of pepsin under acidic conditions [21]. In contrast, the expression of two potential gastric marker genes, mucin-5AC (*MUC5AC*) and the Fc fragment of IgG-binding protein (*FCGBP*), was high in the ♀262-dammed families and ♀412♂387 (Table 2). *MUC5AC* is a major component of the gastric mucus layer [22], while *FCGBP* is functionally related to gel-forming mucins [23], and is a highly expressed gene in the stomach of yellowtail (*Seriola quinqueradiata*) [24]. Furthermore, a trypsinogen (precursor of trypsin) and two solute carrier (SLC) superfamily genes (SLC22A31 and SLC26A9), which are feed-digestion- and nutrient-absorption-related genes, were highly expressed in ♀262-dammed families and ♀412♂387 (Table 2). Trypsin is a proteolytic digestive enzyme that plays an important role in the digestive ability of fish larvae [25], while the SLC superfamily genes encode a series of transporters that play important roles in the absorption of nutrients and ions [26]. It should be noted that murine SLC26A9 is abundantly expressed in the stomach and is an apical HCO_3_^−^ transporter in gastric surface epithelial cells, playing an important role in protecting the gastric epithelium against acidic injury [27].

In contrast, ♀412-dammed families highly expressed immune-related genes, including GTPase IMAP family members [28], perforin [29], glucose-dependent insulinotropic receptor [30], Toll-like receptor 13 [31], and B cell receptor CD22 [32] (Table S1). It should also be noted that the ♀412-dammed families highly expressed transposable-element-related genes (Table S1). We also explored the highly expressed genes in ♀412♂202 (nearly unchanged and small family size) compared with ♀262♂202, ♀262♂432, and ♀412♂387 and identified eight candidate genes. Five of these eight genes also have putative functions in the immune system, including GIMP7, immune-associated nucleotide-binding protein 13 [33], heat shock protein 30 [34], Caspase-1 [35], and E3 ubiquitin-protein ligase RNF144A-A [36] (Table S2).

### 3.3. Correlation with the PG2 Expression Levels at 15 DPH and Larval Survival

**Figure 5:**
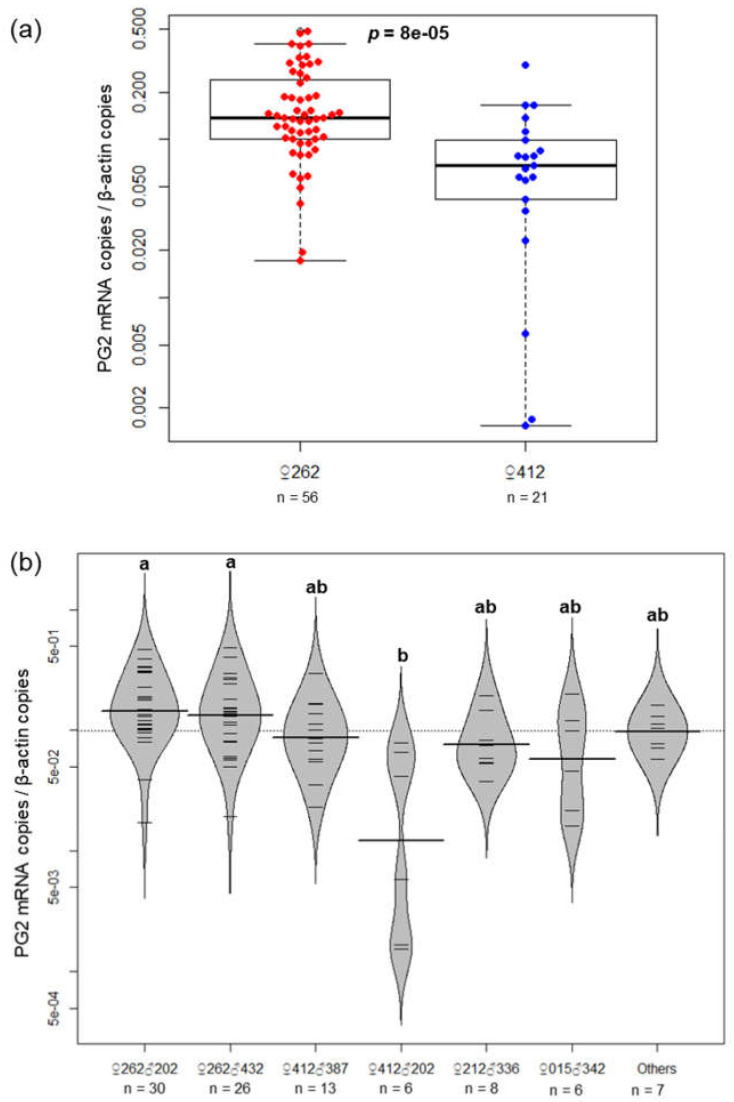
Transcript levels of the *PG2* gene in all genotyped 15 DPH progenies (96 individuals) measured by RT-qPCR. Results are expressed as the ratio of the number of copies of the *PG2* gene to the number of copies of the *β-actin* gene. (**a**) Box plot showing a comparison of the expression level of *PG2* between progenies of ♀262 (low mortality, *n* = 56) and ♀412 (high mortality, *n* = 21). Dots represent individual expression values. (**b**) Bean plots showing expression levels of the *PG2* gene among full-sib families. Others indicate the full-sib families that include no less than three individuals (<*n* = 3). The horizontal lines in each bean refer to individual expression values. The thick black horizontal line within each bean is the mean, while the dashed horizontal line is the overall mean across beans. Different letters represent a significant difference (*p* < 0.05) by one-way analysis of variance with Tukey post hoc analysis.

Because pepsinogen genes showed highly variable expression among families (Table 2 and Figure 4), the gene expression levels of PG2 (the major pepsinogen in PBT [37]) were further analyzed for all 96 genotyped progenies. Comparisons of ♀262- and ♀412-dammed families clearly showed that the *PG2* gene is expressed at significantly higher levels (*p* = 8 × 10^−5^) in ♀262-dammed families (increased family size) at 15 DPH (Figure 5a). Figure 5b demonstrates expression levels of PG2 among full-sib families. The strength of PG2 expression levels mostly corresponded to the family size at 15 DPH (Figure 2 and Figure 5b). The expression of the PG2 gene in the ♀412♂387 family was also higher than that in the ♀412♂202 family, as observed in the oligo-array results (Table 2), but the difference was not statistically significant (Figure 5b). Only two ♀262-dammed families, ♀262♂202 and ♀262♂432, had significantly higher PG2 expression (*p* < 0.05) than that of the ♀412♂202 family (Figure 5b).

## 4. Discussion

A dramatic proportional change in family representations with a strong effect of dams was observed during PBT larval development from 1 to 40 DPH, prominently from 10 to 15 DPH (Figure 2). These results suggested that critical events might be occurring that are responsible for subsequent larval survival at around 15 DPH, particularly in rising ♀262 and declining ♀412 progenies.

Transcriptome analysis of the 15 DPH larvae clearly exhibited that gene expression phenotypes are strongly correlated with the genetic architecture of their parents (Figure 3), with dams having a greater effect than sires, as expected by the parentage assessment (Figure 2). This transcriptome analysis highlighted the importance of the expression levels of genes related to the digestive function of the stomach of 15 DPH larvae for their subsequent survival (Table 2 and Figure 4). This finding was strongly supported by qRT-PCR analysis of PG2 using all genotyped larvae (*n* = 96). The gene expression strength was positively correlated with family survival and was significantly higher in ♀262-dammed families (increased families) than in ♀412-dammed families (declining families; Figure 2 and Figure 5). Although oligo-array results (*n* = 4) showed a high level of PG2 gene expression in ♀412♂387 (Table 2), qRT-PCR results (more quantitative methods and more sample number) did not highlight any statistically difference between the ♀412♂387 and ♀412♂202 families (Figure 5b). The ♀412♂387 family dramatically decreased from 1 to 40 DPH, and most of the larvae died before 40 DPH. Therefore, the results may have been biased by remaining survivors because of higher expression of these genes.

The 15 DPH larvae are at the postflexion stage, where gastric glands begin to form [38,39] and pepsin activity rapidly increases [39,40]. It should be noted that a dramatic increase in the expression of the gastric-function-related genes (Table 2), gastric proton pumps (*ATP4A* and *ATP4B*), pepsinogens *(PG1*, *PG2*, and *PG3*), and potential gastric markers (*MUC5AC*, *FCGBP*, and *SLC26A9*), has been observed from 13 to 15 DPH in a previous transcriptome profiling (same oligo-array used in this study) of PBT early developmental stages (1–25 DPH; unpublished data), suggesting the importance of these genes at 15 DPH. Thus, our transcriptome analysis suggested that the larval stomachs of low-mortality families are more functionally developed than those of high-mortality families at this developmental stage.

In larviculture of PBT, 15 DPH is approximately the timing of the initial feeding of fish larvae in the presence of rotifers and *Artemia* nauplii as prey in the culture tank (Figure 1). Subsequently, marked growth variations are observed, which are related to differences in the initiation of piscivory [14,41] and the use of yolk-sac larvae [42,43]. This growth variation leads to growth-selective mortality, and PBT larvae, which immediately use fish larvae, grow rapidly, and survive in the hatchery tank [14]. These findings suggest that piscivory is a key event for larval survival at around 15 DPH. This study strongly implied that the onset of gastric function in this stage is important for the digestion of large prey, such as fish larvae, which affects subsequent larval survival and is influenced by the genetic background of the parents. In contrast, Sabate et al. [44] reported that the chase behavior of PBT larvae, which is closely related to cannibalistic behavior, was first observed at 14 DPH at the flexion-to-postflexion stages and increased thereafter. Therefore, it is possible that PBT larvae with well-developed gastric functions (i.e., low-mortality families) are capable of cannibalistic attacks on relatively underdeveloped larvae (i.e., high-mortality families) and thereafter induce selective mortality.

Several immune- and transposable-element-related genes were more highly expressed in the ♀412-dammed families than in the ♀262-dammed families at 15 DPH (Table S1). In addition, ♀412♂202 specifically expressed five immune-related genes (Table S2). Transposable elements of eukaryotic genomes have also been reported to be activated by stress and trigger an innate immune response [45,46]. Thus, the regulation of immune function may be more active in the ♀412-dammed families. The possible cause of the negative effects of immune function on survivability may be explained by trade-offs between immune and competing physiological functions [47]. Immune function is an energetically costly physiological activity [47]; thus the energy allocated to gastric functions might be lower in ♀412-dammed families.

Another possibility is that the over-activated immune function might raise survival, compensating the gastric dysfunction, especially in the ♀412♂202 family, which maintained its size throughout the 40 days in this study. An acidic gastric environment sterilizes ingested microorganisms, while they survive through the stomachless fish intestine [48]. Gastric dysfunction larvae might, therefore, suffer opportunistic bacterial infection in their digestive tract, because the intestinal epithelium is a potential port of pathogen entry [49]. The over-activated immune function might alleviate bacterial infection in the gastric dysfunction larvae. Since little is known about the microbiome of tuna larvae, further studies are required to investigate the relationship between the gut microbiome and survival of PBT larvae.

In summary, this study indicated that the functional development of the stomach in PBT larvae during the initial feeding of fish larvae is key to the subsequent survival of PBT larvae in the hatchery tank. In addition, expression levels of gastric-function-related genes (*ATP4A*, *ATP4B*, *PG1*, *PG2*, *PG3*, *MUC5AC*, *FCGBP*, and *SLC26A9*) in this developmental stage might be useful to distinguish between low- and high-mortality families. Recently, RNA-Seq using high-throughput sequencing technology has been developed as an approach for transcriptome analysis [50]. Unlike microarray technology, RNA-Seq can be applied to non-model organisms with no genomic resources. Thus, although our study focused on the larviculture of PBT and used a microarray platform [15], the strategy highlighted herein can be extended to species of interest in aquaculture to find clues to understand the molecular mechanisms linked to variable family survival. This information may be useful for the optimization of breeding technologies in the aquaculture industry.

## Figures and Tables

**Table 1:** Primers and probes used for RT-qPCR assay.

Gene	Forward Primer	Reverse Primer	TaqMan MGB Probe
PG1	AACGAGCTGTACTGGCAGATCA	AGCCACAACCTGACCATTGAC	CAGTGGACAGTGTTACC
PG2	CCGAGGTCACCTTCACTCTGA	GCCAGTTCTGCAACCATAGTAGCT	CTGCATCTGCCTACGTC
PG3	CCACCTACCTGCCCTCTAGTGA	GTGCGGTCGTAGACGGAGTAGT	CTCTGTGGATCTTTG
ATP4A	TCCTCCAAGAGCCACTGTACCT	CACCATGACAACCCTGATACCA	CAGTGATGAAATGTCG
ATP4B	CCATGCCTTGTGTCATCATTAAG	ATTCTCCTGTCCTTCCAGTATGGT	TGAACAGGATCATTGGC
β-actin	GAAATCGCCGCACTGGTT	GCATCGTCTCCGGCAAAT	ATCCGGAATGTGCAAAG

**Table 2:** The top 20 highly expressed genes in three families (♀262♂202, ♀262♂432, and ♀412♂387) compared to the family ♀412♂202 at 15 DPH.

Ranking	Oligo Array Probe ID	Putative Gene Product	♀262♂202/ ♀412♂202	♀262♂432/ ♀412♂202	♀412♂387/ ♀412♂202
1	Ba00000144_g2079	Pepsinogen 3 (PG3)	26.40	22.88	49.55
2	Ba00008844_g23666	Hyaluronidase-1	22.02	26.52	28.03
3	Ba00008341_g23272	Pepsinogen 3 (PG3)	16.78	13.38	27.67
4	Ba00001220_g9569	Potassium-transporting ATPase alpha (ATP4A)	22.39	17.69	16.29
5	isotigB109707_c	Hypothetical protein	16.05	16.33	15.60
6	Ba00000817_g7404	Pepsinogen 1 (PG1)	13.95	14.12	14.04
7	isotigB46117_n	RNA-directed DNA polymerase	17.45	15.52	8.50
8	Ba00002263_g13715	Potassium-transporting ATPase subunit beta (ATP4B)	15.41	10.59	15.01
9	Ba00000128_g1851	Pepsinogen 2 (PG2)	13.06	12.98	13.11
10	isotigB18854_c	Hypothetical protein	7.91	14.33	6.00
11	Ba00000638_g6231	Hypothetical protein	12.22	6.26	5.80
12	Ba00005732_g20720	Hypothetical protein	6.68	8.80	6.61
13	isotigB46118_n	Prostate stem cell antigen	8.78	7.36	4.15
14	Ba00000678_g6517	Solute carrier family 26, member 9 (SLC26A9)	6.47	7.01	5.90
15	Ba00000139_g2005	Trypsinogen	3.81	8.13	6.84
16	Ba00003884_g17895.p2.613-1845	Solute carrier family 22 member 31 (SLC22A31)	5.60	5.27	6.51
17	Ba00000257_g3256	Fc fragment of IgG binding protein (FCGBP)	5.53	6.19	3.57
18	Ba00010561_g24778	ATP-dependent DNA helicase PIF1	4.77	5.98	4.41
19	Ba00004935_g19660	Mucin-5AC (MUC5AC)	4.06	4.36	5.68
20	BaME00000895_g9062	Hypothetical protein	4.94	4.15	4.68

Values indicate the fold-change of expression when compared with the high-mortality family (♀412♂202).

## Data Availability

All raw and processed microarray data were deposited in the DDBJ Genomic Expression Archive under accession number E-GEAD-447.

## Supplementary Materials

The following are available online at https://www.mdpi.com/article/10.3390/cimb43030145/s1.
