# Supplementary material for: Linking Pedigree Information to the Gene Expression Phenotype to Understand Differential Family Survival Mechanisms in Highly Fecund Fish: A Case Study in the Larviculture of Pacific Bluefin Tuna
Source: Curr Issues Mol Biol. 2021 Nov 26;43(3):2098–110. doi: 10.3390/cimb43030145 (PMC8929136; doi:10.3390/cimb43030145)
Supplement: Supplementary file 1 [file cimb-43-00145-s001.zip › cimb-1455562-supplementary.pdf]

current issues in molecular biology

MDPI

Article

Linking Pedigree Information to the Gene Expression Phenotype to Understand Differential Family Survival Mechanisms in Highly Fecund Fish: A Case Study in the Larviculture of Pacific Bluefin Tuna

Motoshige Yasuike [1,*], Kazunori Kumon [2], Yosuke Tanaka [2], Kenji Saitoh [4] and Takuma Sugaya [5]

Table S1 The top 20 highly expressed genes in ♀412-dammed families

Ranking	Oligo array probe ID	Putative gene product	♀412♂387/ ♀262♂202	♀412♂202/ ♀262♂202	♀412♂387/ ♀262♂432	♀412♂202/ ♀262♂432
1	isotigB26288_n	GTPase IMAP family member 7 (GIMAP7)	29.19	17.80	34.79	21.21
2	Ba00008216_g23171	Perforin-1	27.01	13.21	11.86	5.80
3	contigB21468_n	GTPase IMAP family member 4 (GIMAP4)	12.57	8.57	12.46	8.49
4	isotigB149110_c	RNA-directed DNA polymerase from mobile element jockey	12.69	2.62	12.78	2.64
5	Ba00011867_g25412	Reverse transcriptase	6.55	7.72	6.02	7.09
6	isotigB04266_n	Major histocompatibility complex class I-related gene protein (MR1)	6.21	7.17	6.39	7.38
7	isotigB26290_n	GTPase IMAP family member 7 (GIMAP 7)	3.64	11.54	2.68	8.48
8	isotigB142455_c	Hypothetical protein	11.07	2.24	10.82	2.19
9	isotigB04819_n	Glucose-dependent insulinotropic receptor (GIPR)	8.10	4.22	8.96	4.66
10	Ba00010391_g24687	Toll-like receptor 13 (TLR13)	9.00	3.67	9.41	3.84
11	isotigB15872_n	Retrovirus-related Pol poly from transposon	10.23	2.20	10.44	2.25
12	BaME00000660_g7350	Protein PAT1 homolog 2	9.67	2.07	10.70	2.30
13	Ba00001464_g10675	Thyroid peroxidase	10.00	4.20	7.28	3.06
14	isotigB144487_n	Transposon TX1 uncharacterized 149 kDa protein ORF 2	5.21	4.97	7.23	6.90
15	isotigB23166_c	RNA-directed DNA polymerase from mobile element jockey	9.06	2.31	10.07	2.57
16	isotigB04270_n	Major histocompatibility complex class I-related gene protein (MR1)	7.49	7.50	4.45	4.46
17	isotigB13401_c	B-cell receptor CD22	9.75	3.18	8.05	2.63
18	isotigB116404_c	Retrovirus-related Pol poly LINE-1	7.12	5.47	6.22	4.77
19	Ba00000472_g4975	1-phosphatidylinositol 4,5-bisphosphate phosphodiesterase delta-3-A	5.93	2.60	10.45	4.59
20	isotigB142541_c	Hypothetical protein	9.55	2.06	9.74	2.10

Values indicate fold change of expression
The yellow fill and the blue fill indicate immune-related genes and mobile element-related genes, respectively.

Table S2. The highly expressed genes in the family ♀412♂202 than in the three families (♀262♂202, ♀262♂432 and ♀412♂387) at 15 DPH.

Oligo array probe ID	Putative gene product	♀412♂202/ ♀262♂202	♀412♂202/ ♀262♂432	♀412♂202/ ♀412♂437
isotigB26290_n	GTPase IMAP family member 7 (GIMP7)	11.54	8.48	3.17
isotigB176264_n	Immune-associated nucleotide-binding protein 13 (IAN13)	2.66	11.80	4.18
Ba00007516_g22610	heat shock protein 30 (HSP30)	6.37	7.43	2.61
Ba00009804_g24320	reverse transcriptase	5.99	6.74	2.09
Ba00000353_g4052	Caspase-1	3.15	3.46	4.21
Ba00012728_g25757	E3 ubiquitin-protein ligase RNF144A-A	3.70	3.83	2.11
Ba00000386_g4297	Fatty acid-binding protein, intestinal	2.67	3.75	2.01
Ba00000050_g867	REM2- and Rab-like small GTPase 1	2.03	2.07	2.66

Values indicate fold change of expression

The yellow fill and the blue fill indicate immune-related genes and mobile element-related genes, respectively.
